# Relationship between Oxidative Stress Biomarkers and Visual Field Progression in Patients with Primary Angle Closure Glaucoma

**DOI:** 10.1155/2020/2701539

**Published:** 2020-08-05

**Authors:** Shengjie Li, Mingxi Shao, Yingzhu Li, Xiaojuan Li, Yani Wan, Xinghuai Sun, Wenjun Cao

**Affiliations:** ^1^Clinical Laboratory, Eye & ENT Hospital, Fudan University, Shanghai, China; ^2^Eye Institute and Department of Ophthalmology, Eye & ENT Hospital, Fudan University, Shanghai, China; ^3^State Key Laboratory of Medical Neurobiology, Institutes of Brain Science, Fudan University, Shanghai, China; ^4^Key Laboratory of Myopia, Chinese Academy of Medical Sciences, Shanghai, China; ^5^NHC Key Laboratory of Myopia, Fudan University, Shanghai, China

## Abstract

**Purpose:**

To investigate the serum changes of oxidative stress markers and the relationship between these factors and visual field (VF) progression in patients with primary angle closure glaucoma (PACG).

**Methods:**

A case-control and a prospective cohort study. A total of 94 patients with PACG and 89 normal controls were enrolled. Furthermore, 94 PACG subjects were followed up for at least two years (once every six months). All participants were evaluated for serum levels of superoxide dismutase (SOD), total antioxidant status (TAS), hydrogen peroxide (H_2_O_2_), malondialdehyde (MDA), glutathione peroxidase, glutathione reductase, and detailed eye and systematic examination. Binary logistic regression analysis and Cox regression analysis were performed.

**Results:**

The serum levels of SOD and TAS in the PACG group were significantly lower than those in the control group (*p* < 0.001). Meanwhile, PACG subjects had significantly higher levels of MDA and H_2_O_2_ than the normal control subjects (*p* < 0.001). Serum levels of TAS (OR = 0.773, 95%CI = 0.349 − 0.714, *p* < 0.001), SOD (OR = 0.975, 95%CI = 0.955 − 0.995, *p* < 0.001), MDA (OR = 1.155, 95%CI = 1.080 − 1.235, *p* < 0.001), and H_2_O_2_ (OR = 1.216, 95%CI = 1.142 − 1.295, *p* < 0.001) were independent risk/protective factors for PACG. TAS levels (HR = 0.041, 95%CI = 0.008–0.218, *p* < 0.001), SOD levels (HR = 0.983, 95%CI = 0.971–0.994, *p* = 0.003), and MDA levels (HR = 1.010, 95%CI = 1.001–1.018, *p* = 0.015) at baseline were associated with visual field progression. Kaplan–Meier curves reveal that patients with TAS < 0.95/SOD < 143/MDA > 12 had a significantly higher percentage of PACG progression (*p* < 0.05).

**Conclusions:**

Decreased levels of TAS and SOD as well as increased levels of MDA at baseline were associated with VF progression in patients with PACG. These findings suggest that oxidative stress was involved in the onset and development of PACG.

## 1. Introduction

Primary glaucoma represents a group of diseases defined by characteristically elevated intraocular pressure (IOP), visual dysfunction, and optic nerve head cupping and is one of the major causes of irreversible blindness worldwide [1, 2]. The global prevalence of glaucoma in those between the ages of 40 and 80 is roughly 3.54% [3], and primary angle closure glaucoma (PACG) is the most common type of glaucoma—roughly 1.5 times more common than primary open-angle glaucoma (POAG) in China [4]. For PACG, in which the angle closure is a fundamental pathologic process, the precise mechanisms involved in progressive retinal ganglion cell (RGC) death are yet to be determined [5].

Recently, the gradual accumulation of evidence has suggested that the imbalance of oxidative stress may play an important role in the RGC damage of PACG [6–10]. Goyal et al. [7] performed a case-control study which reported a significant increase in superoxide dismutase (SOD) and glutathione peroxidase (GPX) activities, which were found in the aqueous humor of PACG patients compared to cataract patients. Adav et al. [6] suggested that the critical mediators of oxygen homeostasis and neuronal function in the aqueous humor were significantly dysregulated in disease, strongly implicating oxidative stress responses in PACG-associated nerve damage. Moreover, Chang et al. [9] performed a small case-control study and found that the concentration of malondialdehyde (MDA) in PACG patients was significantly higher than that of the control subjects. Furthermore, our previous study also reported that the serum level of uric acid, a major antioxidant molecule, was significantly lower in PACG [11] and POAG [12] subjects than that in the control subjects. These results may suggest that evaluating oxidative stress may help in understanding the course of PACG, and oxidative stress damage might be a relevant target for both glaucoma prevention and therapy.

However, with respect to PACG, the results are mainly derived from small-scale case-control studies, and no longitudinal study has yet assessed the relationship between the level of oxidative stress biomarkers and rates of visual field (VF) loss. In the present study, we aim to perform a case-control study and prospective cohort study to validate the significance of oxidative stress biomarkers as predictors on VF progression in patients with PACG.

## 2. Materials and Methods

### 2.1. Patients

This study was conducted at the Department of Ophthalmology and Visual Sciences, Eye and Ear, Nose, and Throat (ENT) Hospital of Fudan University, Shanghai, China. Approval from the Institutional Review Board/Ethics Committee was obtained from the Ethics Committee of the Eye and ENT Hospital, and the study adhered to the principles of the Declaration of Helsinki. Informed consent was obtained from all subjects.

From June 2016 to December 2017, PACG subjects were recruited from the Department of Ophthalmology and Visual Sciences at the Eye and ENT Hospital of Fudan University. Age and sex-matched normal controls were consecutively recruited from subjects who participated in annual health screenings during the study period. This case-control study was performed to compare the levels of oxidative stress-related factors between PACG and normal subjects as well as to establish novel biochemical risk factors for PACG.

Furthermore, to assess whether oxidative stress-related factors could be used to predict VF progression in PACG subjects, all PACG subjects were visited once every six months to keep the investigators up-to-date on the progression of PACG (the minimum follow-up period was set to 24 months). Assuming a VF endpoint in 45% of patients by the end of two years, the hazard ratio was 0.55. Fixing a type 1 error at 5% (two-sided) and power at 80%, 88 patients will be needed for the analysis.

### 2.2. Diagnostic and Inclusion Criteria

PACG was diagnosed by a glaucoma specialist. Previously described diagnostic criteria were used to diagnose PACG [13–17]. PACG was diagnosed based on narrow anterior chamber angles with glaucomatous optic neuropathy and corresponding VF loss. VF loss was determined by including a cluster of three or more nonedge contiguous points on the pattern deviation plot, which did not cross the horizontal meridian, and had a probability of less than 5% of being present in age-matched controls (one of which was less than 1%); it had an abnormal standard deviation pattern with a *p* < 0.05 occurring in the normal population and fulfilled the following test reliability criteria: less than 20% fixation losses, less than 15% false positives, and/or less than 15% false negatives. Additionally, PACG was diagnosed in eyes with narrow angles, elevated IOP (IOP > 21 mm Hg), having at least 180° of angle-closure obliterating the pigmented segment of the trabecular meshwork, whether synechial, appositional, segmented, or continuous, and in cases where the degree of peripheral anterior synechiae was too extensive to be managed by laser peripheral iridotomy.

The inclusion criteria for PACG subjects and normal subjects were as follows: IOP < 21 mm Hg for normal subjects and IOP > 21 mm Hg for PACG, absence of any secondary glaucoma or any other eye disease that could potentially affect visual acuity or the VF, absence of any intraocular surgery within the previous two months, absence of diabetes mellitus and hypertension, and absence of any systemic diseases, including acute infectious diseases, metabolic syndrome, autoimmune disease, or cancer. No PACG patients were treated with LPI or trabeculectomy prior to entering the study and during the follow-up period. Most of the PACG patients received topical glaucoma medications.

In this study, 61 PACG subjects (other type of glaucoma = 7, cataract = 18, other eye diseases = 5, cancer = 2, intraocular surgery = 3, metabolic syndrome = 5, autoimmune disease = 2, acute infectious diseases = 4, refused to participate = 2, lost to follow − up = 6, diabetes mellitus = 4, and hypertension = 3) and 39 normal control subjects (IOP > 21 = 4, cataract = 4, VCDR > 0.6 = 4, other eye diseases = 3, cancer = 2, metabolic syndrome = 3, autoimmune disease = 4, acute infectious diseases = 5, other systemic diseases = 5, diabetes mellitus = 2, and hypertension = 3) were excluded based on the diagnostic and inclusion criteria. Finally, a total of 94 subjects with PACG and 89 normal controls were enrolled in this study.

### 2.3. Examination

Clinical data were obtained from PACG patients who underwent a standardized ophthalmic examination conducted by a glaucoma specialist. This examination included the assessment of the anterior chamber angle by gonioscopy (Haag-Streit, Bern, Switzerland) and a three-time IOP measurement using Goldmann applanation tonometry (Haag-Streit, Bern, Switzerland) which was then averaged. The eye's fundus was analyzed using a digital retinal camera (TRC-NW200, Topcon). An A-scan ultrasound (A-Scan Pachymeter, Ultrasonic, Exton, PA, USA) was used to measure the central corneal thickness (CCT), axial length (AL), and anterior chamber depth (ACD). The vertical cup-disk ratio (VCDR) was evaluated by two doctors based on an analysis of the fundus photos and then averaged. Each normal control subject underwent preliminary ophthalmic examinations, which included gonioscopy, a slit-lamp examination, and IOP, all performed by glaucoma specialists.

Medical examinations were performed by the respective specialty physicians for all subjects at the Eye and ENT Hospital of Fudan University. Medical examinations, including the assessment of electrocardiograms, X-rays, liver function, blood glucose, infectious diseases, renal function, blood pressure, heart rate, IOP-lowering medication, body temperature, height, and weight, were performed for all subjects. The body mass index (BMI) was calculated as the weight in kilograms divided by the height in meters squared. Information about drinking and smoking was also collected. Drinking was defined as >3 times per week and >6 per month (current or former); smoking was defined as >1 cigarette per day and >6 per month (current or former) [18].

### 2.4. Laboratory Detection

Laboratory testing was performed at the Department of Clinical Laboratory, Eye and ENT Hospital of Fudan University. Blood samples were obtained in the morning after subjects fasted for eight hours via standard venipuncture of the veins in the antecubital fossae (anterior elbow veins). The tubes were centrifuged for 10 minutes at 3000 r/min. The blood samples were processed within one hour of collection, and the serum was frozen at -80°C. GPX serum levels (Pyrogallol Substrate Method, ZhongTuo biotechnology, Shandong, China), glutathione reductase (GR, glutathione substrate method, ZhongTuo biotechnology, Shandong, China), SOD (UV enzymatic method, JiuQiang biotechnology, Beijing, China), and total antioxidant status (TAS, colorimetry method, JiuQiang biotechnology, Beijing, China) were measured using a commercially available kit by Roche Diagnostics Cobas 8000 (Mannheim, Germany).

The serum samples were subjected to an MDA assay as described in the lipid peroxidation MDA assay kit (Beyotime, Nantong, China). The MDA concentration of each sample was detected by multimode microplate readers (Biotek SynergyH1, USA) at 532 nm. The serum samples were subjected to a hydrogen peroxide (H_2_O_2_) assay as described in the hydrogen peroxide assay kit (Beyotime, Nantong, China). The H_2_O_2_ concentration of each sample was detected by multimode microplate readers (Biotek SynergyH1, USA) at 560 nm.

### 2.5. VF Analysis

The previously described methods were used for the VF analysis as follows [13]. The Glaucoma Department of the Eye and ENT Hospital of Fudan University performed perimetry on the glaucoma subjects unless they were unable to see any light with both eyes open or suffered from an eye infection. The mean deviation (MD) and mean sensitivity of the VFs were measured with an Octopus-automated perimeter. All patients underwent a minimum of five VF tests. After taking into account the learning effect of the VF tests, the results of the first two tests were excluded. Only reliable (a false positive/negative below 15% and a reliability factor below 20%) and compatible VF results were included. Each patient had a minimum of one reliable VF test. VF testing was completed at baseline and every six months during the follow-up. The previously described methods [19, 20] were used to determine the functional PACG VF progression according to the event-based analysis modified for Octopus perimetry [21] as follows (satisfying at least one of the following criteria): (1) developing a new scotoma of at least three nonedge points worsening ≥ 5 dB or one nonedge point worsening ≥ 10 dB; (2) a cluster of ≥3 nonedge points with ≥10 dB deteriorating in a preexisting scotoma; (3) developing a new cluster of ≥3 nonedge points with 15 degrees around a preexisting scotoma; and (4) worsening of the global MD value by ≥2 dB/y. The first eye of each patient to progress was included for the analysis. If both eyes progressed at the same time, the greater progressed eye was included during the follow-up period.

### 2.6. Statistical Analysis

All analyses were performed using version 19.0 of the Statistical Package for the Social Sciences (SPSS Inc., Chicago, IL, USA). The figures were created using GraphPad Prism 6 (La Jolla, CA, USA). The results are presented as a mean ± SD. Normality was assessed using a Kolmogorov-Smirnoff test.

In the case-control study, an independent Student's *t*-test, Mann-Whitney *U* test, Fisher exact test, and *χ*^2^ test were used to compare patient characteristics between the groups as appropriate. The odds ratios (OR) and their corresponding 95% CI were determined using binary logistic regression models that included covariates for age, gender, BMI, systolic blood pressure (SBP), diastolic blood pressure (DBP), smoking (yes/no), drinking (yes/no), the number of topical glaucoma medications, and IOP. Furthermore, the associations between IOP and VCDR with oxidative stress biomarkers were analyzed using a Spearman analysis.

In the cohort study, univariate and multivariate Cox regression analyses were used to analyze the association between baseline oxidative stress-related factors with PACG progression. Furthermore, Cox proportional hazards models were used to obtain hazard ratios (HR) and to identify baseline factors that predicted which subjects would be classified into the nonprogressing PACG group during the follow-up period. Based on the median baseline levels of TAS, SOD, and MDA, the PACG subjects were categorized into upper and lower subgroups (TAS < 0.95, TAS > 0.95; SOD > 143, SOD < 143; MDA > 12, MDA < 12). The cumulative incidence of nonprogressing PACG according to the oxidative stress levels was assessed using Kaplan-Meier plots, and the log-rank test was used to assess differences between the curves. A value of *p* < 0.05 was considered statistically significant.

## 3. Results

### 3.1. Case-Control Study

According to the screening criteria, a total of 94 subjects with PACG (male = 37, female = 57) and 89 normal controls (male = 34, female = 55) were enrolled in this study. Only one eye was randomly selected if both eyes suffered from PACG at baseline.  Table 1 summarizes the demographic and ocular characteristics of the PACG and control groups.

There was no statistical difference in the mean age, sex distribution, BMI, SBP, DBP, smoking, and drinking between PACG and control subjects (*p* > 0.05) (Table 1). The mean serum levels of SOD and TAS in the PACG group were significantly lower than those in the control group (*p* < 0.001) (Table 1). Meanwhile, PACG subjects had significantly higher levels of MDA and H_2_O_2_ than the normal control subjects (*p* < 0.001) (Table 1).

**Figure 1:**
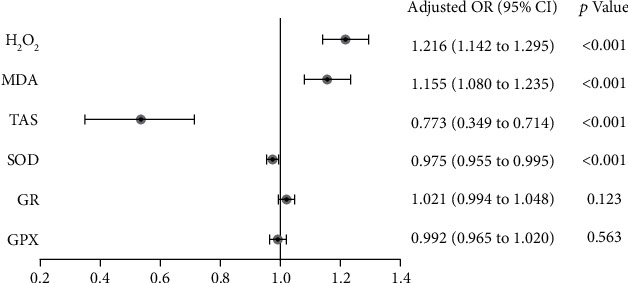
Logistic regression analyses to identify risk factors for primary angle closure glaucoma in comparison to the control subjects, after adjusting for age, gender, BMI, SBP, DBP, smoking (yes/no), drinking (yes/no), number of topical glaucoma medications, and IOP. SOD: superoxide dismutase; TAS: total antioxidant status; H_2_O_2_: hydrogen peroxide; MDA: malondialdehyde; GPX: glutathione peroxidase; GR: glutathione reductase.

Binary logistic regression analyses identified the risk factors for PACG in comparison to the control subjects (Figure 1). After adjusting for age, gender, BMI, SBP, DBP, smoking (yes/no), drinking (yes/no), the number of topical glaucoma medications, and IOP, the analyses revealed that TAS (OR = 0.773, 95%CI = 0.349–0.714, *p* < 0.001), SOD (OR = 0.975, 95%CI = 0.955–0.995, *p* < 0.001), MDA (OR = 1.155, 95%CI = 1.080–1.235, *p* < 0.001), and H_2_O_2_ (OR = 1.216, 95%CI = 1.142–1.295, *p* < 0.001) were independent risk/protective factors for PACG.

The Spearman analysis showed a statistically significant negative correlation between TAS and VCDR (*r* = 0.449, *p* < 0.001). In addition, there was a statistically significant positive correlation between the MDA levels and VCDR (*r* = 0.319, *p* = 0.02). Moreover, there was no statistically significant correlation between IOP and VCDR with the other oxidative stress biomarkers.

### 3.2. Cohort Study

A total of 94 subjects with PACG were included. Among these patients, 43 (45.74%) showed progression of glaucoma as measured by VF.  Table 2 summarizes the demographic and disease characteristics of PACG subjects with and without VF progression at baseline.

At baseline, there were no significant between-group differences in gender, BMI, SBP, DBP, smoking, drinking, the number of topical glaucoma medications, IOP, VCDR, CCT, AL, MD, GPX, GR, or H_2_O_2_ (*p* > 0.05). The mean serum levels of TAS (*p* < 0.001) and SOD (*p* = 0.001) in the progression group were significantly lower than the nonprogressing group, and the mean level of MDA was significantly higher in the progression group than the nonprogressing group (*p* = 0.007) (Table 2).

To assess the value of baseline oxidative stress-related factors associated with the progression of PACG, we performed a Cox proportional hazards regression analysis (Table 3). A higher baseline TAS (HR = 0.041, 95%CI = 0.008–0.218, *p* < 0.001), higher baseline SOD levels (HR = 0.983, 95%CI = 0.971–0.994, *p* = 0.004), higher baseline MDA levels (HR = 1.013, 95%CI = 1.001–1.016, *p* = 0.003), and females (HR = 2.228, 95%CI = 1.013–4.897, *p* = 0.046) were associated with glaucoma progression as measured by VF in a multivariate Cox analysis after adjusting for age, gender, BMI, SBP, DBP, smoking (yes/no), drinking (yes/no), the number of topical glaucoma medications, IOP, VCDR, CCT, AL, and ACD.

**Figure 2:**
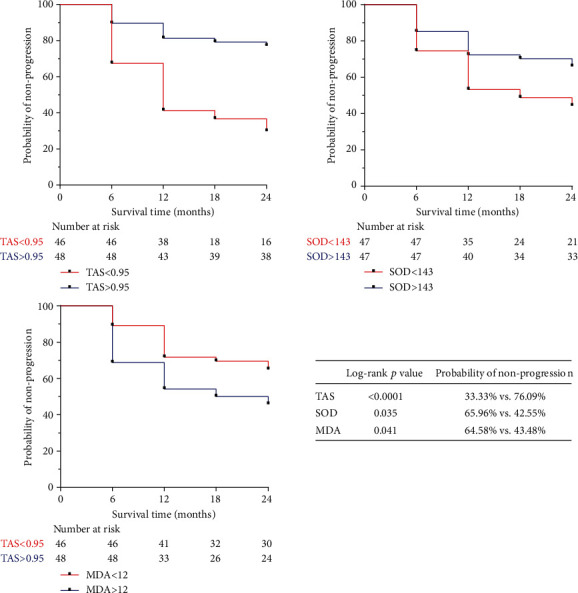
Kaplan-Meier survival curves showing the effects of oxidative stress-related factors on visual field progression, derived from Cox univariate analyses. Survival curves of the upper and lower subgroup were compared using log-rank tests. Factors analyzed included superoxide dismutase (SOD), total antioxidant status (TAS), and malondialdehyde (MDA).

Based on the median baseline levels of TAS, SOD, and MDA, the PACG subjects were categorized into upper and lower subgroups (TAS < 0.95, TAS > 0.95; SOD > 143, SOD < 143; MDA > 12, MDA < 12).  Figure 2 shows the Kaplan-Meier survival curves. A survival analysis indicated that patients withTAS < 0.95had a significantly higher percentage of PACG progression (log-rank test*p* < 0.0001) and patients withSOD < 143had a significantly higher percentage of PACG progression (log-rank test*p* = 0.035). Patients with MDA > 12 also had a significantly higher percentage of PACG progression (log-rank test *p* = 0.041).

BMI: body mass index; SBP: systolic blood pressure; DBP: diastolic blood pressure; IOP: intraocular pressure; VCDR: vertical cup-disc ratio; CCT: central corneal thickness; ACD: anterior chamber depth; AL: axial length; MD: visual field mean deviation; NA: not applicable; SOD: superoxide dismutase; TAS: total antioxidant status; H_2_O_2_: hydrogen peroxide; MDA: malondialdehyde; GPX: glutathione peroxidase; GR: glutathione reductase.

## 4. Discussion

In this relatively large-scale case-control and prospective cohort study, we demonstrated that decreased TAS, decreased SOD, increased MDA, and increased H_2_O_2_ are independent risk factors for PACG. Furthermore, our multivariate analyses have shown that low baseline serum TAS and SOD are risk factors independent from other factors and could accelerate the progression of PACG. Meanwhile, high baseline serum MDA is also a risk factor independent from other factors and could accelerate the progression of PACG. These results may suggest that an imbalance of oxidative stress was involved in the onset and development of PACG, and the above oxidative stress-related biomarkers could be useful markers for predicting the progression of patients with PACG.

To date, there has been increasing evidence to suggest that an imbalance in the oxidative stress system may be involved in the pathogenesis of glaucoma. Several studies have investigated the level of oxidative stress-related markers in the aqueous humor [7, 22] and peripheral blood [9, 23, 24] of patients with glaucoma, as well as the expression of oxidative stress-related markers in humans [25, 26] and an experimental glaucoma model's ocular tissues [27]. Engin et al. [28] reported that the serum levels of TAS, SOD, and GPX decreased and serum levels of MDA increased in patients with glaucoma, which provides compelling evidence for imbalance in the oxidative stress system in the peripheral blood of glaucoma. Nucci et al. [22] suggested that there were higher levels of MDA and lower levels of TAS in the aqueous humor of patients with glaucoma, and McElnea et al. [26] found that intracellular reactive oxygen species production-MDA was increased in the lamina cribrosa from glaucomatous human donor eyes compared to normal human donor eyes. The above two studies provide compelling evidence for imbalance in the oxidative stress system in ocular glaucoma. Furthermore, in the experimental glaucoma model, increased immunostaining for MDA and 3-nitrotyrosine occurred in RGCs and other neurons in acute glaucoma [27].

Although numerous studies have investigated the relationship between oxidative stress-related markers and glaucoma, almost all focus on open-angle glaucoma and exfoliation glaucoma. For open-angle glaucoma and exfoliation glaucoma, the imbalance of oxidative stress has already been reported by many studies and was further confirmed by meta-analyses [29, 30]. Only four studies have examined the relationship of oxidative stress with angle closure glaucoma, of which two studies were for TAS [31, 32], one for MDA [9], and one for SOD and GPX [7]. Regarding TAS, the previously reported data were conflicting. Abu-Amero et al. [32] reported that the mean TAS value was incredibly similar in PACG patients (1.0 ± 0.22) compared to the controls (0.97 ± 0.43) (*p* = 0.345). Furthermore, Mousa et al. [31] have shown that the mean level of TAS was significantly lower among PACG subjects (0.98 ± 0.41) than the controls (1.1 ± 0.22). In the present study, the mean level of TAS in PACG and normal controls was 0.98 ± 0.17 and 1.22 ± 0.16, respectively. The mean serum levels of TAS in the PACG group were significantly lower. Interestingly, the level of TAS in PACG patients in the present study was similar to the results reported by Mousa et al. [31] and Abu-Amero et al. [32]. Therefore, our results concerning TAS concentrations are credible and comparable with previous studies. Moreover, Chang et al. [9] reported in a smaller study (*n* = 50) the higher levels of MDA in the serum of patients with PACG, and Goyal et al. [7] reported in 30 patients that a significant increase in SOD activities was found in the aqueous humor of PACG patients. In this relative large-scale study, we have found that the mean serum levels of SOD in the PACG group were significantly lower, and PACG subjects had significantly higher levels of MDA and H_2_O_2_ than the normal control subjects. Our results concerning MDA concentrations are consistent with previous studies. However, the SOD results were contradictory, which may be due to different specimen sources (serum vs. aqueous humor). These results suggest that the oxidative stress system appears to be disordered in patients with PACG.

The possible role of the oxidative stress system in the progression of glaucoma has not been previously elucidated. A better understanding of the serum levels of oxidative stress markers and their possible role in PACG may be clinically useful in the management of the disease. To the best of our knowledge, this is the first prospective cohort study which focuses on evaluating the relationship with oxidative stress biomarkers with PACG progression. In the present study, low baseline serum levels of TAS, SOD, and high MDA could accelerate the progression of PACG. Limited data are available in the literature regarding the association of oxidative stress markers with PACG progression, but there has been increasing evidence showing that TAS, SOD, and MDA levels could serve as biomarkers to predict the progression or survival of different types of other diseases [33–36]. For example, Chen et al. [33] performed a community-based cohort study of 2224 participants reporting that increased activity of SOD was independently associated with lower all-cause mortality in older women. A meta-analysis of five prospective studies showed that a significant inverse association was found between dietary TAS and all-cause mortality (combined effect size = 0.62, 95%CI = 0.60–0.64), cancer (combined effect size = 0.81, 95%CI = 0.75–0.88), and cardiovascular disease mortality (combined effect size = 0.71, 95%CI = 0.63–0.82) [34].

For eye diseases, Rautiainen et al. [35] have shown that dietary TAS was inversely associated with the risk of age-related cataract in a population-based prospective cohort of middle-aged and elderly women. Kuppan et al. [37] reported that the systemic levels of MDA increased with the progression of type 2 diabetes mellitus without diabetic retinopathy to diabetic retinopathy. Moreover, several studies have suggested that antioxidant treatment can decrease oxidative stress and improve neuron survival in glaucoma [38–41]. For example, Ramdas et al. [38] performed a prospective population-based study which indicated that dietary intake of low antioxidative property nutrients appears to be associated with an increased risk of open-angle glaucoma. Lee et al. [39] reported that coenzyme Q10 promoted RGC survival by approximately 29% by inhibiting oxidative stress-mediated mitochondrial alteration in a mouse model of glaucoma. However, there has been no study reporting the association between oxidative stress markers and progression of glaucoma.

The possible role of oxidative stress damage in the progression of glaucoma has not been clarified. Oxidative stress damage reflects a disbalance between prooxidants and antioxidants. TAS is applied to reflect the overall antioxidant status. Antioxidant enzymes, including SOD and GPX, are the first lines of defense against oxidative stress and act by scavenging potentially damage free radical moieties [42, 43]. H_2_O_2_ has been considered a powerful prooxidant agent, and the presence of oxidative stress has been demonstrated through markers of lipid peroxidation MDA, which was the end product of the primary reactions of oxidative stress. In the present study, the SOD and TAS levels decreased and the MDA and H_2_O_2_ levels increased in the PACG patients. Thus, in glaucoma, the existence of excess prooxidants cannot be effectively eliminated by antioxidants. Oxidative stress can inflict damage to nucleic acids, proteins, and lipids, which leads to the damage of trabecular meshwork cells and RGC [44]. Moreover, we also reported that decreased TAS and SOD and increased MDA levels at baseline are significant predictors of VF progression in patients with PACG. Thus, oxidative stress was a risk factor in the onset/development of glaucoma. One of the mechanisms is the direct neurotoxicity to RGC induced by oxidative stress [45]. Another potential mechanism is that oxidative stress stimulates neuroinflammation [46] to accelerate the death of RGC by activating receptor-mediated inflammation signaling [47], stimulating the antigen presentation [48], and complementing dysregulation [14, 49]. Oxidative stress can result in a greater extent of systemic inflammation by the secretion and release of cytokines [46], which in turn accelerate the progression of glaucoma. For the most part, the body's antioxidant capacity was mainly a result of the daily dietary intake of nutrients, and the eating habits of each person were relatively stable. Thus, we speculate that the concentration of prooxidant and antioxidant agents was relatively stable for each person. Essentially, baseline oxidative stress markers can predict the progression of glaucoma.

Elevated IOP is a risk factor for glaucomatous progression. Patients with PACG with higher IOP show higher rates of progression compared to patients with relatively lower IOP [50, 51]. In this study, there was no statistical difference in the mean IOP between the progression group (20.60 ± 10.30 mm Hg) and the no progression group (22.59 ± 17.10 mm Hg), and the mean level of IOP in the no progression group seems a little high. The reason for this inconsistency may be as follows: (1) in this study, the vast majority of cases had the disease for a long duration and received glaucoma medications. The results may have been affected either by behavioral changes consequent to the knowledge of their disease status or by any kind of treatment; (2) moreover, older age and severity are also risk factors for glaucomatous progression [50, 51], which may also affect the progression of glaucoma. In this study, the mean age in the progression group was significantly higher than that in the no progression group (56 versus 61.07 years old, *p* = 0.036), and the level of mean deviation in the progression group seems a little high compared to the no progression group; and (3) Cioffi et al. [52] also reported that some patients with high IOP do not develop glaucoma and some patients worsen despite adequate IOP control.

Although the present study is the first to focus on the evaluation of serum TAS, SOD, and MDA levels, and their relationship to with progression of PACG, we acknowledge that it has some limitations. First, it was conducted in a single center and the participants were all Chinese. Therefore, our results may not be generalizable to other populations. Second, the progression of glaucoma is not just visual fields, and disc changes, especially when the visual field loss is slight. However, spectral domain optical coherence tomography was not performed during the follow-up period, which may bring some confounding. Third, cardiovascular disease also plays an important role in glaucoma progression [53, 54], and this could also impact the level of oxidative stress biomarkers. Although patients with diabetes mellitus and hypertension were excluded, some patients with potential cardiovascular complications may have been included in this study. Finally, despite adjusting for many potential confounders, including age, gender, BMI, SBP, DBP, smoking, drinking, and the number of topical glaucoma medications, drug therapies may have changed during the course of the study which would have an impact on the results.

## 5. Conclusions

Taken together, we found that (1) higher serum MDA levels, lower serum TAS, and SOD levels were associated with a higher risk of PACG; and (2) the serum levels of TAS, SOD, and MDA at baseline were associated with the progression of PACG as measured by the VF and could be useful markers for predicting the progression of patients with PACG. These findings indicated that an imbalance in the oxidative stress system was involved in the onset and development of glaucoma, and oxidative stress might be a relevant target for both glaucoma prevention and therapy.

## Figures and Tables

**Table 1:** Demographics and clinical characteristics.

Variable	Control group (*n* = 89)	PACG group (*n* = 94)	*p* value
Age (year)	57.03 ± 8.35	58.32 ± 13.51	0.437^&^
Gender (male/female)	34/55	37/57	0.872^∗^
BMI (m^2^/kg)	22.94 ± 3.18	22.52 ± 2.97	0.348^&^
SBP (mm Hg)	126.29 ± 9.10	124.52 ± 13.82	0.306^&^
DBP (mm Hg)	73.99 ± 7.52	73.30 ± 9.46	0.589^&^
Smoking (yes/no)	20/69	23/71	0.750^∗^
Drinking (yes/no)	22/67	24/70	0.899^∗^
Number of topical glaucoma medications, *n*			
0	—	4	—
1-2	—	33	—
>2	—	57	—
IOP (mm Hg)	13.04 ± 3.23	21.68 ± 14.36	<0.001^&^
VCDR	—	0.63 ± 0.23	—
CCT (mm)	—	545.34 ± 43.80	—
ACD (cm)	—	1.99 ± 0.51	—
AL (cm)	—	22.65 ± 1.41	—
MD (dB)	—	10.98 ± 7.42	—
MS (dB)	—	17.34 ± 7.45	—
GPX (U/L)	57.36 ± 9.67	56.30 ± 12.18	0.517^&^
GR (U/L)	59.79 ± 11.57	62.43 ± 12.98	0.148^&^
SOD (U/mL)	169.00 ± 38.44	140.99 ± 28.35	<0.001^&^
TAS	1.22 ± 0.16	0.98 ± 0.17	<0.001^&^
MDA (umol/l)	5.57 ± 4.49	30.69 ± 35.99	<0.001^&^
H_2_O_2_ (umol/l)	8.20 ± 6.25	18.03 ± 10.93	<0.001^&^

^&^Independent-samples *t*-test; ^#^Mann-Whitney *U* test; ^∗^*χ*^2^ test. BMI: body mass index; SBP: systolic blood pressure; DBP: diastolic blood pressure; IOP: intraocular pressure; VCDR: vertical cup-disc ratio; CCT: central corneal thickness; ACD: anterior chamber depth; AL: axial length; MD: visual field mean deviation; MS: visual field mean sensitivity; SOD: superoxide dismutase; TAS: total antioxidant status; H_2_O_2_: hydrogen peroxide; MDA: malondialdehyde; GPX: glutathione peroxidase; GR: glutathione reductase.

**Table 2:** Baseline demographic and disease characteristics of PACG subjects with and without visual field progression.

Variable	No progression (*n* = 51)	Progression (*n* = 43)	t value	*p* value
Age (year)	56.00 ± 14.32	61.07 ± 12.07	2.134	0.036^&^
Gender (male/female)	21/30	16/27	0.154	0.695^∗^
BMI (m^2^/kg)	22.83 ± 2.77	22.15 ± 3.19	1.112	0.269^&^
SBP (mm Hg)	124.06 ± 12.18	125.07 ± 15.78	0.349	0.728^&^
DBP (mm Hg)	73.67 ± 8.83	72.86 ± 10.26	0.409	0.684^&^
Smoking (yes/no)	10/41	13/30	1.425	0.233^∗^
Drinking (yes/no)	15/36	9/34	0.883	0.347^∗^
Number of topical glaucoma medications, *n*				
0	2	2		
1-2	20	13		
>2	35	22	0.382	0.938^∗^
Visual acuity (logMAR)	0.17 ± 0.10	0.19 ± 0.09	1.063	0.290^&^
IOP (mm Hg)	22.59 ± 17.10	20.60 ± 10.30	0.667	0.506^&^
VCDR	0.64 ± 0.23	0.62 ± 0.23	0.277	0.783^&^
CCT (mm)	547.09 ± 38.29	543.39 ± 49.68	0.391	0.697^&^
ACD (cm)	2.10 ± 0.59	1.86 ± 0.36	2.387	0.019^&^
AL (cm)	22.85 ± 1.69	22.42 ± 0.93	1.455	0.149^&^
MS(dB)	18.19 ± 7.06	16.36 ± 7.85	1.185	0.239^&^
MD (baseline, dB)	10.19 ± 7.12	11.92 ± 7.75	1.125	0.264^&^
MD (6 months, dB)	10.52 ± 7.07	12.64 ± 7.75	1.395	0.166^&^
MD (12 months, dB)	10.78 ± 7.09	12.87 ± 7.51	1.386	0.169^&^
MD (18 months, dB)	11.05 ± 7.09	13.63 ± 7.70	1.685	0.095^&^
MD (24 months, dB)	11.38 ± 7.10	14.17 ± 7.62	1.835	0.070^&^
GPX (U/L)	56.92 ± 13.70	55.55 ± 10.19	0.541	0.590^&^
GR (U/L)	63.05 ± 14.64	61.69 ± 10.80	0.503	0.616^&^
SOD (U/mL)	151.07 ± 26.04	132.49 ± 27.64	3.333	0.001^&^
TAS	1.05 ± 0.12	0.90 ± 0.18	4.557	<0.001^&^
MDA (umol/l)	21.55 ± 25.57	41.55 ± 43.21	2.779	0.007^&^
H_2_O_2_ (umol/l)	16.89 ± 10.88	19.37 ± 10.97	1.094	0.277^&^

^&^Independent-samples *t*-test; ^#^Mann-Whitney *U* test; ^∗^*χ*^2^ test. BMI: body mass index; SBP: systolic blood pressure; DBP: diastolic blood pressure; IOP: intraocular pressure; VCDR: vertical cup-disc ratio; CCT: central corneal thickness; ACD: anterior chamber depth; AL: axial length; MD: visual field mean deviation; MS: visual field mean sensitivity; SOD: superoxide dismutase; TAS: total antioxidant status; H_2_O_2_: hydrogen peroxide; MDA: malondialdehyde; GPX: glutathione peroxidase; GR: glutathione reductase.

**Table 3:** Factors associated with progression of PACG as measured with visual field using Cox proportional hazards analysis.

Variable	Univariate		Multivariate	
	HR (95% CI)	*p* value	HR (95% CI)	*p* value
Age (year)	1.018 (0.994-1.043)	0.148	NA	NA
Female	1.965 (1.007-3.833)	0.048	2.228 (1.013-4.897)	0.046
BMI (m^2^/kg)	0.954 (0.858-1.061)	0.389	NA	NA
SBP (mm Hg)	1.003 (0.981-1.026)	0.772	NA	NA
DBP (mm Hg)	0.993 (0.962-1.026)	0.684	NA	NA
Smoking (yes/no)	1.418 (0.739-2.719)	0.293	NA	NA
Drinking (yes/no)	0.724 (0.347-1.510)	0.389	NA	NA
IOP (mm Hg)	0.993 (0.969-1.018)	0.597	NA	NA
VCDR	0.776 (0.183-3.296)	0.731	NA	NA
CCT (mm)	0.998 (0.991-1.005)	0.598	NA	NA
ACD (cm)	0.445 (0.198-0.996)	0.049	0.483(0.196-1.190)	0.114
AL (cm)	0.804 (0.595-1.086)	0.155	NA	NA
MD (dB)	1.018 (0.979-1.059)	0.365	NA	NA
GPX (U/L)	0.993 (0.968-1.019)	0.583	NA	NA
GR (U/L)	0.994(0.970-1.018)	0.606	NA	NA
SOD (U/mL)	0.984 (0.975-0.995)	0.005	0.983 (0.971-0.994)	0.003
TAS	0.055 (0.013-0.238)	<0.001	0.041 (0.008-0.218)	<0.001
MDA (umol/l)	1.010 (1.003-1.017)	0.008	1.010 (1.001-1.018)	0.015
H_2_O_2_ (umol/l)	1.013 (0.989-1.038)	0.274	NA	NA

## Data Availability

The data used to support the findings of this study are available from the corresponding author upon request.

## References

[1] Quigley H. A. (2011). Glaucoma. *Lancet*.

[2] Sun X., Dai Y., Chen Y. (2017). Primary angle closure glaucoma: What we know and what we don't know. *Progress in Retinal and Eye Research*.

[3] Tham Y.-C., Li X., Wong T. Y., Quigley H. A., Aung T., Cheng C.-Y. (2014). Global prevalence of glaucoma and projections of glaucoma burden through 2040: a systematic review and meta-analysis. *Ophthalmology*.

[4] Song P., Wang J., Bucan K., Theodoratou E., Rudan I., Chan K. Y. (2017). National and subnational prevalence and burden of glaucoma in China: a systematic analysis. *Journal of Global Health*.

[5] Jonas J. B., Aung T., Bourne R. R., Bron A. M., Ritch R., Panda-Jonas S. (2017). Glaucoma. *Lancet*.

[6] Adav S. S., Wei J., Qian J., Gan N. Y., Yip L. W. L., Sze S. K. (2019). Aqueous humor protein dysregulation in primary angle-closure glaucoma. *International Ophthalmology*.

[7] Goyal A., Srivastava A., Sihota R., Kaur J. (2014). Evaluation of oxidative stress markers in aqueous humor of primary open angle glaucoma and primary angle closure glaucoma patients. *Current Eye Research*.

[8] Cantó A., Olivar T., Romero F. J., Miranda M. (2019). Nitrosative stress in retinal pathologies: review. *Antioxidants*.

[9] Chang D., Sha Q., Zhang X. (2011). The evaluation of the oxidative stress parameters in patients with primary angle-closure glaucoma. *PLoS One*.

[10] Saccà S. C., Cutolo C. A., Ferrari D., Corazza P., Traverso C. E. (2018). The eye, oxidative damage and polyunsaturated fatty acids. *Nutrients*.

[11] Li S., Shao M., Tang B., Zhang A., Cao W., Sun X. (2017). The association between serum uric acid and glaucoma severity in primary angle closure glaucoma: a retrospective case-control study. *Oncotarget*.

[12] Li S., Shao M., Li D., Tang B., Cao W., Sun X. (2019). Association of serum uric acid levels with primary open-angle glaucoma: a 5-year case-control study. *Acta Ophthalmologica*.

[13] Li S., Shao M., Cao W., Sun X. (2019). Association between pretreatment serum uric acid levels and progression of newly diagnosed primary angle-closure glaucoma: a prospective cohort study. *Oxidative Medicine and Cellular Longevity*.

[14] Li S., Chen Y., Shao M., Tang L., Sun X., Cao W. (2017). Association of plasma complement C3 levels with primary angle-closure glaucoma in older women. *Investigative Ophthalmology & Visual Science*.

[15] Li S., Shao M., Wan Y., Tang B., Sun X., Cao W. (2019). Relationship between ocular biometry and severity of primary angle-closure glaucoma: relevance for predictive, preventive, and personalized medicine. *The EPMA Journal*.

[16] Li S., Zhang H., Shao M. (2020). Association Between 17-*β*-Estradiol and Interleukin-8 and Visual Field Progression in Postmenopausal Women with Primary Angle Closure Glaucoma. *American Journal of Ophthalmology*.

[17] Hodapp E., Parrish R. K., Anderson D. R. (1993). *Clinical decisions in glaucoma*.

[18] Li S., Li D., Shao M., Cao W., Sun X. (2018). Decreased serum levels of complement C3 reflect complement system dysregulation in patients with primary open-angle glaucoma: results from a pilot study. *Journal of Glaucoma*.

[19] Chen Y., Qiu C., Qian S. (2018). Lack of association of rs1192415 in TGFBR3-CDC7 with visual field progression: a cohort study in Chinese open angle Glaucoma patients. *Frontiers in Genetics*.

[20] South East Asia Glaucoma Interest Group (2008). *Asia Pacific Glaucoma Guidelines*.

[21] Naghizadeh F., Holló G. (2014). Detection of early glaucomatous progression with octopus cluster trend analysis. *Journal of Glaucoma*.

[22] Nucci C., Di Pierro D., Varesi C. (2013). Increased malondialdehyde concentration and reduced total antioxidant capacity in aqueous humor and blood samples from patients with glaucoma. *Molecular Vision*.

[23] Erdurmuş M., Yağcı R., Atış Ö., Karadağ R., Akbaş A., Hepşen I. F. (2011). Antioxidant status and oxidative stress in primary open angle glaucoma and pseudoexfoliative glaucoma. *Current Eye Research*.

[24] Mumcu U. Y., Kocer I., Ates O., Alp H. H. (2016). Decreased paraoxonase1 activity and increased malondialdehyde and oxidative DNA damage levels in primary open angle glaucoma. *International Journal of Ophthalmology*.

[25] Fernández-Durango R., Fernández-Martínez A., García-Feijoo J. (2008). Expression of nitrotyrosine and oxidative consequences in the trabecular meshwork of patients with primary open-angle glaucoma. *Investigative Ophthalmology & Visual Science*.

[26] McElnea E. M., Quill B., Docherty N. G. (2011). Oxidative stress, mitochondrial dysfunction and calcium overload in human lamina cribrosa cells from glaucoma donors. *Molecular Vision*.

[27] Chen T., Gionfriddo J. R., Tai P.-Y., Novakowski A. N., Alyahya K., Madl J. E. (2015). Oxidative stress increases in retinas of dogs in acute glaucoma but not in chronic glaucoma. *Veterinary Ophthalmology*.

[28] Engin K. N., Yemişci B., Yiğit U., Ağaçhan A., Coşkun C. (2010). Variability of serum oxidative stress biomarkers relative to biochemical data and clinical parameters of glaucoma patients. *Molecular Vision*.

[29] Tang B., Li S., Cao W., Sun X. (2019). The association of oxidative stress status with open-angle glaucoma and exfoliation glaucoma: a systematic review and meta-analysis. *Journal of Ophthalmology*.

[30] Benoist d’Azy C., Pereira B., Chiambaretta F., Dutheil F. (2016). Oxidative and anti-oxidative stress markers in chronic glaucoma: a systematic review and meta-analysis. *PLoS One*.

[31] Mousa A., Kondkar A. A., Al-Obeidan S. A. (2015). Association of total antioxidants level with glaucoma type and severity. *Saudi Medical Journal*.

[32] Abu-Amero K. K., Azad T. A., Mousa A., Osman E. A., Sultan T., Al-Obeidan S. A. (2014). Total antioxidant level is correlated with intra-ocular pressure in patients with primary angle closure glaucoma. *BMC Research Notes*.

[33] Mao C., Yuan J.-Q., Lv Y.-B. (2019). Associations between superoxide dismutase, malondialdehyde and all-cause mortality in older adults: a community-based cohort study. *BMC Geriatrics*.

[34] Parohan M., Anjom-Shoae J., Nasiri M., Khodadost M., Khatibi S. R., Sadeghi O. (2019). Dietary total antioxidant capacity and mortality from all causes, cardiovascular disease and cancer: a systematic review and dose-response meta-analysis of prospective cohort studies. *European Journal of Nutrition*.

[35] Rautiainen S., Lindblad B. E., Morgenstern R., Wolk A. (2014). Total antioxidant capacity of the diet and risk of age-related cataract: a population-based prospective cohort of women. *JAMA Ophthalmology*.

[36] Yepes-Calderón M., Sotomayor C., Gomes-Neto A. (2019). Plasma malondialdehyde and risk of new-onset diabetes after transplantation in renal transplant recipients: a prospective cohort study. *Journal of Clinical Medicine*.

[37] Kuppan K., Mohanlal J., Mohammad A. M. (2019). Elevated serum OxLDL is associated with progression of type 2 diabetes mellitus to diabetic retinopathy. *Experimental Eye Research*.

[38] Ramdas W. D., Wolfs R. C. W., Kiefte-de Jong J. C. (2012). Nutrient intake and risk of open-angle glaucoma: the Rotterdam Study. *European Journal of Epidemiology*.

[39] Lee D., Shim M. S., Kim K.-Y. (2014). Coenzyme Q10 inhibits glutamate excitotoxicity and oxidative stress-mediated mitochondrial alteration in a mouse model of glaucoma. *Investigative Ophthalmology & Visual Science*.

[40] Inman D. M., Lambert W. S., Calkins D. J., Horner P. J. (2013). *α*-Lipoic acid antioxidant treatment limits glaucoma-related retinal ganglion cell death and dysfunction. *PLoS One*.

[41] Ko M.-L., Peng P.-H., Hsu S.-Y., Chen C.-F. (2010). Dietary deficiency of vitamin E aggravates retinal ganglion cell death in experimental glaucoma of rats. *Current Eye Research*.

[42] Harman D. (2006). Free radical theory of aging: an update: increasing the functional life span. *Annals of the New York Academy of Sciences*.

[43] Ung L., Pattamatta U., Carnt N., Wilkinson-Berka J. L., Liew G., White A. J. R. (2017). Oxidative stress and reactive oxygen species: a review of their role in ocular disease. *Clinical Science*.

[44] Sies H. (2015). Oxidative stress: a concept in redox biology and medicine. *Redox Biology*.

[45] Doi K., Uetsuka K. (2011). Mechanisms of mycotoxin-induced neurotoxicity through oxidative stress-associated pathways. *International Journal of Molecular Sciences*.

[46] Yang X., Hondur G., Tezel G. (2016). Antioxidant treatment limits neuroinflammation in experimental glaucoma. *Investigative Ophthalmology & Visual Science*.

[47] Tezel G., Luo C., Yang X. (2007). Accelerated aging in glaucoma: immunohistochemical assessment of advanced glycation end products in the human retina and optic nerve head. *Investigative Ophthalmology & Visual Science*.

[48] Tezel G., Yang X., Luo C., Peng Y., Sun S. L., Sun D. (2007). Mechanisms of immune system activation in glaucoma: oxidative stress-stimulated antigen presentation by the retina and optic nerve head glia. *Investigative Ophthalmology & Visual Science*.

[49] Tezel G., Yang X., Luo C. (2010). Oxidative stress and the regulation of complement activation in human glaucoma. *Investigative Ophthalmology & Visual Science*.

[50] Ernest P. J., Schouten J. S., Beckers H. J., Hendrikse F., Prins M. H., Webers C. A. (2013). An evidence-based review of prognostic factors for glaucomatous visual field progression. *Ophthalmology*.

[51] Zhang X., Parrish R. K., Greenfield D. S. (2019). Predictive Factors for the Rate of Visual Field Progression in the Advanced Imaging for Glaucoma Study. *American Journal of Ophthalmology*.

[52] Cioffi G. A. (2005). Ischemic model of optic nerve injury. *Transactions of the American Ophthalmological Society*.

[53] Leske M. C., Heijl A., Hyman L., Bengtsson B., Dong L., Yang Z. (2007). Predictors of Long-term Progression in the Early Manifest Glaucoma Trial. *Ophthalmology*.

[54] Chan T. C. W., Bala C., Siu A., Wan F., White A. (2017). Risk factors for rapid glaucoma disease progression. *American Journal of Ophthalmology*.

